# Selective Solar Harvesting Windows for Full‐Spectrum Utilization

**DOI:** 10.1002/advs.202201738

**Published:** 2022-06-05

**Authors:** Weihong Li, Chongjia Lin, Gan Huang, Jun Hur, Baoling Huang, Shuhuai Yao

**Affiliations:** ^1^ Department of Mechanical Engineering City University of Hong Kong Tat Chee Avenue Kowloon 999077 Hong Kong; ^2^ Department of Mechanical and Aerospace Engineering Hong Kong University of Science and Technology Clear Water Bay Kowloon 999077 Hong Kong; ^3^ Institute of Microstructure Technology Karlsruhe Institute of Technology Eggenstein‐Leopoldshafen 76344 Germany

**Keywords:** full‐spectrum utilization, solar harvesting window, transparent photovoltaic, transparent solar absorber

## Abstract

Smart windows can selectively regulate excess solar radiation to reduce heating and cooling energy consumption in the built environment. However, the inevitable dissipation of ultraviolet and near‐infrared into waste heat results in inefficient solar utilization. Herein, a dual‐band selective solar harvesting (SSH) window is developed to realize full‐spectrum utilization. A transparent photovoltaic, converting ultraviolet into electricity, and a transparent solar absorber, converting near‐infrared into thermal energy, are integrated and coupled with a ventilation system to extract heat for indoor use. Compared with common transparent photovoltaics, the SSH window increases solar harvesting efficiency up to threefold while maintaining a considerable visible transmittance. Simulations suggest that the SSH window, besides generating electricity, delivers energy savings by over 30% higher than common smart windows. This is the first integration of transparent photovoltaic and transparent solar absorber into a window, which may open up a new avenue for the development of energy‐efficient buildings.

## Graphical abstract

A selective solar harvesting window is designed as a photovoltaic‐thermal collecting system coupled with a ventilation system to extract heat for indoor use. The integration of the transparent photovoltaic and transparent solar absorber enables a dual‐band selectivity for full‐spectrum solar utilization, opening up a new avenue for the development of energy‐efficient buildings.

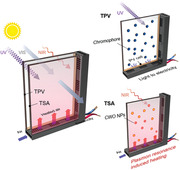

## 1 Introduction

The Intergovernmental Panel on Climate Change (IPCC) alarms an extreme urgency of climate change by reducing the global warming allowance from 2 to 1.5 °C and calls for global endeavor to efficient energy conservation and carbon emission reduction.^[^ ^1^ ^]^ Building sector accounts for around 40% of total energy consumption in developed countries,^[^ ^2^ ^]^ while around half of the energy is consumed for the heating, ventilation, and air conditioning (HVAC) systems^[^ ^2^, ^3^ ^]^. Windows represent the major source of heat gain or loss as well as of visual and thermal discomfort; however, they are regarded as the least energy‐efficient sector of building envelope. Therefore, various energy‐saving windows have been developed and most works focus on spectrally selective optical coatings,^[^ ^4^ ^]^ chromogenic coatings based on electrochromic, photochromic, and thermochromic phenomena,^[^ ^5^ ^]^ and integration of near‐ultraviolet (UV) solar cells with electrochromic window.^[^ ^6^ ^]^ Overall, these windows enable control of luminous and near‐infrared (NIR) light transmission and regulation of the solar heating effect.^[^ ^7^ ^]^ Although they relieve the burden of indoor cooling and reduce building energy consumption, these windows inevitably dissipate a large portion of solar energy in the UV and NIR bands into waste heat, particularly in hot seasons, degrading building energy efficiency, and intensifying the urban heat island effect. Therefore, it is desirable to develop windows that can harvest the dissipated solar energy either in the form of electricity or thermal energy for further utilization in buildings.

One widely‐used solution to harvest the full‐spectrum solar energy is a photovoltaic‐thermal (PV/T) collecting system.^[^ ^8^ ^]^ However, for window applications, both photovoltaic and thermal collector have to be visibly transparent, and thus conventional PV/T designs cannot meet the demand. Emerging transparent photovoltaics (TPVs) absorb a portion of solar irradiance to generate electricity and have a desirable visible transmittance.^[^ ^9^ ^]^ They are categorized into visible light‐absorption‐type TPVs^[^ ^10^ ^]^ and luminescent solar concentrator (LSC) type TPVs.^[^ ^11^ ^]^ Since they merely absorb a limited portion of UV or NIR light, current TPVs suffer from low solar harvesting efficiency. To enable a broadband solar absorption, transparent solar absorbers (TSAs) are utilized to harvest the complementary portion of UV or NIR light by converting the harvested light into thermal energy. Common TSAs, e.g., photonic metamaterials^[^ ^12^ ^]^ and multilayer thin‐film structures,^[^ ^13^ ^]^ however, show low visible transmittance or low temperature rises under solar illumination, or require complicated fabrication manners and high costs, and thus are not ideal for the transparent solar–thermal systems. Until recently, a few highly efficient and cost‐effective TSAs have been reported.^[^ ^14^ ^]^ We developed a cesium‐doped tungsten trioxide (CWO) based TSA that achieved a visible transmittance of up to 82% and an absorption of NIR of >90%,^[^ ^14a^ ^]^ offering a great opportunity for transparent solar–thermal conversion. Later, a power‐generating window was developed by coupling the CWO‐based TSA with thermoelectric devices and demonstrated a conversion efficiency comparable to state‐of‐the‐art TPVs.^[^ ^14b^ ^]^ However, such a power‐generating window suffered from low thermal‐to‐electricity conversion efficiency as well as incompactness and aesthetic problems, as bulk thermoelectric devices were incorporated in the system. Up to date, despite these efforts, full‐spectrum solar utilization, i.e., selectively transmitting visible light and harvesting both UV and NIR light, is still unavailable for window applications. In addition, as a practical consideration, it is essential to develop a simple architecture with fewer material restrictions and lower cost.

**Figure 1:**
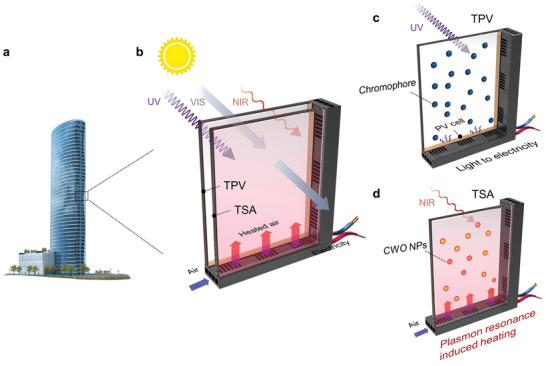
Concept of the selective solar harvesting (SSH) window. a) lass‐encrusted high‐rise buildings. b) Schematic of the SSH window showing the transparent photovoltaic (TPV), transparent solar absorber (TSA), and flowing air system. Schematic of the c) TPV part and d) TSA part.

In this work, we develop a dual‐band selective solar harvesting (SSH) window composed of a TPV and a TSA. The SSH window acts as a transparent photovoltaic/thermal (PV/T) system that converts UV into electricity by the TPV and NIR into thermal energy by the TSA while simultaneously transmits visible light (**Figure** **1**). The harvested thermal energy is extracted by ventilated air to provide indoor space heating in cold seasons or abate indoor cooling loading in hot seasons. We demonstrated that the SSH window has a visible transmittance of 42%, achieves a solar‐electricity conversion efficiency of 0.75%, and a solar–thermal conversion efficiency of 24% with a ventilated air temperature rise of 10 °C. Moreover, compared with common TPVs, SSH window increases solar harvesting efficiency by up to threefold. Simulations by Energy Plus suggest that the SSH window can save the annual HVAC energy consumption by up to 61.5% compared with the normal glass, in addition to the generated electricity that accounts for up to 19.1% of the annual energy saving amount. The SSH window represents the first reported transparent PV/T system, and has superior advantages in processability, cost‐effectiveness, and commercial adaptability, which may initiate a new design concept for energy‐efficient buildings.

## 2 Results and Discussion

### 2.1 Concept of the Dual‐Band SSH Window

A solar harvesting window is designed to selectively harvest the complementary solar energy other than visible light for indoor utilization, through a synergistic integration of a TPV that converts the solar radiation in the UV band into electricity and a TSA that converts the solar energy in the NIR band into thermal energy. The schematic of the SSH window is shown in Figure 1b. A luminescent solar concentrator type TPV (Figure S1, Supporting Information) is utilized as the exterior window (Figure 1c), which mainly absorbs UV light from CuInS2/ZnS quantum dots and emits light to opaque PVs that are located at the edge of the transparent substrate for electricity generation. Concurrently, the TSA is utilized as the interior window (Figure 1d), which transmits visible light and absorbs the remaining UV and NIR to produce heat, owing to the localized surface plasmon resonances of CWO nanoparticles (NPs).^[^ ^14a^ ^]^ Subsequently, thermal energy is mostly extracted by the ventilated air within the gap for various purposes such as indoor space heating in cold seasons.

### 2.2 Optical‐Electrical‐Thermal Properties of the SSH Window

**Figure 2:**
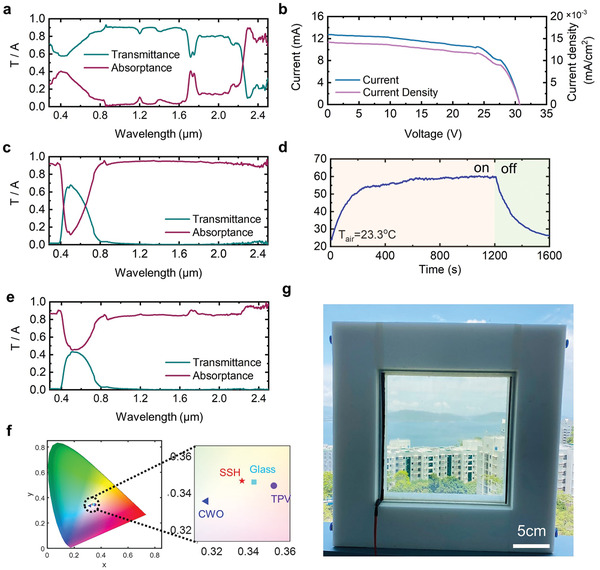
Properties of window components. a) Spectra of the TPV. b) Current–voltage curves of the TPV. c) Spectra of the TSA. d) Temperature response of the TSA. e) Spectra of the SSH window. f) CIELAB color space diagram of the SSH window. g) Prototype of the SSH window.

With respect to the properties of TPV, we measured the transmittance spectra (*T*) and absorption spectra (*A*) shown in **Figure** **2**a. The TPV shows a high absorptance of UV light (37.3%) and a high transmittance of visible light (72.3%) and NIR light (85.4%). The TPV was characterized under a standard solar simulator of AM1.5G (1000 W m^−2^) at 25 °C and its current–voltage (*I–V*) response is shown in Figure 2b, giving an open–circuit voltage (*V*_oc_) of 30 V, a short‐circuit current density (*J*_sc_) of 0.015 mA cm^−2^. In regard to the TSA, we developed a solution‐based process, i.e., spraying a mixed solution of colloidal CWO and BTA NPs and acrylic resin on a glass substrate (Figure S2, Supporting Information). The cross‐sectional scanning electron microscope (SEM) image (Figure S3, Supporting Information) shows the selective photothermal coating on the substrate. The impacts of mass fraction of CWO NPs and absorber thickness on the photothermal efficiency have been extensively quantified,^[^ ^14a^ ^]^ and thereby, here, we adopted the optimal design. Figure 2c shows the spectra of the CWO coated glass, exhibiting an exceptional spectral selectivity, i.e., absorption of the UV and NIR and transmission of the visible light. Quantitatively, the mean solar absorptance (calculation methods can be found in Note S1, Supporting Information) of the sample is 64.5%, and visible transmittance *T*_v_ is 71.8%. We further characterized the heating behavior of the coating under solar illumination (details can be found in “Experimental Section”). The temperature rose rapidly within 3 min upon illumination and reached a plateau up to 60 °C (Figure 2d) and dropped back to room temperature after the light was turned off. We have demonstrated that the TSA exhibits remarkable improvement in photothermal efficiency and visible transparency compared with previous studies.^[^ ^14a^ ^]^ After assembling the SSH window, we further characterized its overall spectra (Figure 2e) and observed a remarkable dual‐band spectral selectivity, i.e., high visible transmittance of 41.2% and high UV and NIR absorption of over 90%. To better assess the practical application of our SSH window, we evaluated the aesthetic parameter of CIELAB color coordinates,^[^ ^13d^ ^]^ which is the figure of merit that indicates the presence or absence of color and displays both chromaticity and brightness values, per the International Commission on Illumination (CIE) (calculation methods can be found in Note S2, Supporting Information). The SSH window is depicted by CIE 1931 XYZ coordinates in Figure 2f, along with a CWO glass, a TPV glass, and a normal glass, and is found to have a color coordinate close to the normal glass. A prototype with dimensions of L (30 cm) × H (30 cm) × W (2.4 cm) was made by assembling a TPV facing exterior and a TSA facing interior, and it showed a substantial visible transmittance (Figure 2g).

### 2.3 SSH Window Field Demonstration

**Figure 3:**
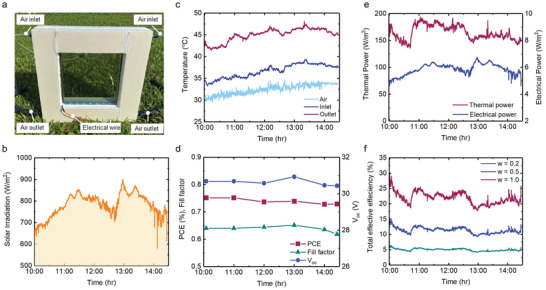
Field solar harvesting tests. a) Field test setup. b) Solar irradiance and c) Inlet and outlet air temperature and ambient temperature on October 11, 2021at Hong Kong. d) Electrical performances of the SSH window. e) Calculated thermal power and electrical power generations. f) Total effective efficiency with different weighting coefficients *w*.

We further built a field set‐up (**Figure** **3**a) to quantify the solar harvesting efficiency of the SSH window. The measurement was conducted from 10:00 am to 14:30 pm on October 11th, 2021. The solar irradiance (*G*) of this period is shown in Figure 3b. We monitored the air temperatures at the inlet (*T*_fi_) and outlet (*T*_fo_) of the SSH window and the ambient air temperature (Figure 3c) and obtained the average temperature difference (Δ*T* = *T*_fo_–*T*_fi_) to be around10 °C. During the test period, we recorded the *I–V* curve of the SSH window at six typical time points and plotted the power conversion efficiency (*PCE*), fill factor (*FF*), and *V*_OC_ with time in Figure 3d. Here, *PCE* indicates the percentage of the solar energy shining on the window that is converted into electricity and is calculated by (*J*_sc_,•*V*_oc_,•*FF*)/G.^[^ ^15^ ^]^, which applies to TPVs and luminescent solar concentrators. Under varying solar irradiance, the *V*_oc_ and *FF* remained almost constant and the *PCE* was found constant around 0.75%. With the temperature data, air mass flow rate *ṁ*, PCE, and *G*, we further calculated the harvested thermal power *w*_th_, thermal efficiency *η*_th_, electrical power *w*_ele_, and electrical efficiency *η*_ele_ (calculation methods can be found in Note S3, Supporting Information). When calculating the thermal efficiency *η*_th_, we did not consider the pumping power of ventilation system, because it was negligible compared with the generated electricity. Figure 3e shows that the generated electrical power is around 6 W m^−2^ and the thermal power is around 150 W m^−2^, which is 25 times of the generated electrical power, suggesting that the harvesting thermal power is of primary importance for building‐integrated solar energy harvesting windows. Here, we consider the equivalence of the thermal power and electrical power and provide an overall estimation to the SSH window's solar energy conversion efficiency with a total effective efficiency,^[^ ^16^ ^]^ i.e., *η*_tot_ = *η*_ele_ + *w*•*η*_th_, where *w* is a weight coefficient that ranges from 0 to 1 and represents the worth of thermal energy relative to that of electricity. The value of *w* can be based on a thermodynamic value (e.g., via the second‐law arguments or heat engine conversion efficiencies), a cost value (e.g., through a price ratio of heat/electricity), or a ratio of environmental benefits (e.g., displaced or mitigated emissions). We plotted the total effective efficiency with three *w* values of 0.2, 0.5, and 1.0. As *w* increases, the thermal energy becomes more valuable (relative to electricity) and the total effective efficiency significantly increases. At an extreme scenario of *w* = 1.0, the thermal energy is equivalent to the generated electricity, e.g., electric heater, and thus the SSH window produces the highest efficiency of around 24%, which is comparable to conventional non‐transparent PV/T systems.^[^ ^17^ ^]^.This demonstrates that the SSH window holds great potential for building‐integrated solar energy harvesting and indoor space heating.

### 2.4 Indoor Temperature Regulation Test

**Figure 4:**
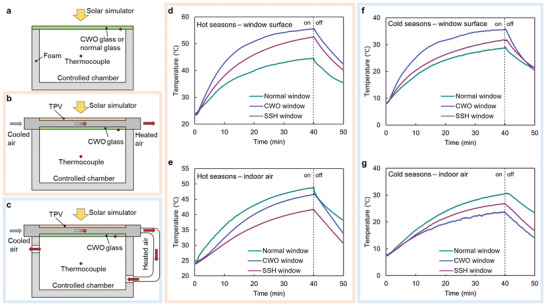
Indoor thermal management tests. Schematic of a) normal window or CWO window, b) SSH window with air ventilated outside in simulated hot seasons, and c) SSH window with air‐ventilated indoor in simulated cold seasons. d) Window surface temperature in simulated hot seasons. e) Indoor air temperature in simulated hot seasons. f) Window surface temperature in simulated cold seasons. g) Indoor air temperature in simulated cold seasons.

Besides the TPV and TSA, an air ventilation system, which collects the thermal energy and delivers the heat to regulate the indoor temperature, is another essential component of the SSH window. We further quantified the indoor temperature regulation capacity by monitoring the inner temperatures of three chambers equipped with different types of windows (i.e., a normal glass window, a CWO window, and an SSH window) under different environmental conditions (**Figure** **4**a–c; Figure S4, Supporting Information). A solar simulator was used as a light source and the ambient/chamber temperature was controlled to simulate hot seasons and cold seasons. In hot seasons, the heated air is directed to outside to reduce indoor cooling loading (Figure 4b), while in cold seasons, the heated air is delivered into the chamber for indoor space heating (Figure 4c). We measured the thermo‐responsive behaviors by monitoring the window surface temperature and indoor temperature versus time. In hot seasons, the ambient temperature was set at 28 °C and the solar simulator power was *G* = 1 kW m^−2^. The surface temperature of the CWO window was the highest due to strong photothermal efficiency, while that of the SSH window was lower due to the convective heat loss from the flowing air (Figure 4d). Thanks to the continuous heat removal from the flowing air, the indoor temperature with the SSH window was lower than those with CWO window and normal window (Figure 4e), which was favorable for reducing indoor cooling loading. In cold seasons, the ambient temperature was set at 8 °C and the solar simulator power was *G* = 0.8 kW m^−2^. The window surface temperatures followed similar trends as those in hot seasons (Figure 4f). However, since the heated air was directed to the chamber, the SSH window showed a higher indoor temperature than that of CWO window (Figure 4g), which was favorable for reducing indoor heating loading. By changing the air flow path, we can switch the SSH window to different energy‐saving modes and regulate the indoor temperature to cope with different seasons. The dynamic switch between the cooling and heating modes makes the SSH window more adaptive to weather changes than the static CWO window or normal windows.

### 2.5 Thermal and Overall Efficiency of the SSH Window

**Figure 5:**
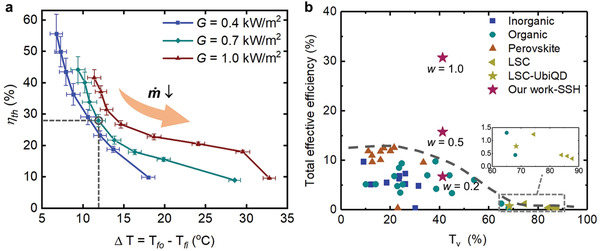
Thermal and overall efficiency. a) Thermal efficiency of the SSH window as a function of the inlet and outlet air temperature difference (Δ*T* = *T*_fo_–*T*_fi_) at a given solar irradiance. Error bars represent plus or minus one standard error of the mean of three repeated measurements. b) Comparison of the total effective efficiency and visible transmittance *T*_v_ for the SSH window and the previous transparent photovoltaics.

To thoroughly evaluate the thermal efficiency of the SSH window, we explored a matrix of air mass flow rate and solar irradiance, monitored the air temperature rise Δ*T*, and further calculated the thermal efficiency *η*_th_. The test matrix was composed of *G* at 0.4, 0.7, and 1.0 kW m^−2^ and *ṁ* ranging from 0.012 to 0.178 kg min^−1^. We plotted the *η*_th_ versus Δ*T* under different solar irradiance, as Δ*T* is directly associated with the indoor heating demand. We observe, in **Figure** **5**a, that the *η*_th_ shows a monotonically decreasing trend with Δ*T* and is elevated as *G* increases. Note that the increasing of Δ*T* is accompanied with the decreasing of *ṁ* due to longer heating time. Assuming *ṁ* approaches infinity, the *η*_th_ and Δ*T* will approach unit and zero, respectively. On the contrary, assuming *ṁ* approaches zero that corresponds to a static SSH window, the *η*_th_ will approach zero (no thermal energy harvesting) while the Δ*T* will converge to a certain value that is determined by the photothermal efficiency. Assuming that ambient air is 8 °C in cold seasons (e.g., in tropical cities), the air from HVAC systems needs to be heated by 12 °C to provide indoor thermal comfort according to ASHRAE Standard 55—2017.^[^ ^18^ ^]^ Therefore, we selected this condition by a circled point (Δ*T* = 12 °C, *G* = 0.7 kW m^−2^) in Figure 5a and obtained *η*_th_ = 30%. This value was used for the total effective efficiency calculation.

We further summarized the solar harvesting efficiency data of common TPVs and compared with our SSH window in terms of the total effective efficiency *η*_tot_ versus visible transmittance *T*_v_ (Figure 5b). We calculated the *η*_tot_ of the SSH window by setting *η*_th_ = 30%. Note that the *η*_tot_ is equal to the *η*_ele_ for common TPVs without solar–thermal conversion component. Most TPVs show η_tot_ below 12% and *T*_v_ below 35%. By comparison, the SSH window obtains its peak *η*_tot_ of 30.8% when the weight coefficient *w* = 1 with *T*_v_ of 41.2%, which is remarkably higher than other TPVs. The *η*_tot_ of the SSH window drops to 15.8% and 6.8% when the weight coefficient *w* = 0.5 and 0.2, respectively. Note that when *w* is >0.36, which is the conversion factor of the thermal power plant,^[^ ^16^ ^]^ the SSH window would outperform the most efficient TPV ever reported^[^ ^19^ ^]^ (*η*_tot_ = *η*_ele_ = 12.6%, *T*_v_ = 21.5%). We believe, in most scenarios, our SSH window will serve as one of the most efficient candidates for building‐integrated solar harvesting devices.

### 2.6 Energy Consumption Simulations

**Figure 6:**
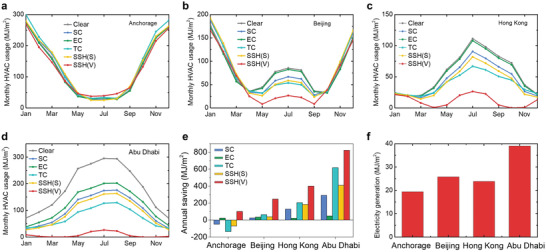
Annual energy saving in different climate zones by simulation. Monthly HVAC energy consumption in a) Anchorage, b) Beijing, c) Hong Kong, d) Abu Dhabi with clear window, solar controlled (SC) window, electrochromic (EC) window, thermochromic (TC) window, and SSH window without (S) and with ventilation (V). e) Annual energy saving of different windows on the basis of the clear window. f) Electricity generation of the SSH window.

To estimate the potential energy saving by our SSH windows, we used EnergyPlus to simulate the energy consumptions with the indoor temperature controlled between 20 and 26 °C by an HVAC system, following the recommendation by the U.S. DOE.^[^ ^20^ ^]^ Four typical cities, e.g., Anchorage, Beijing, Hong Kong, and Abu Dhabi, were chosen to evaluate the monthly HVAC energy consumption across different climate zones. The simulations were conducted with an ventilated SSH window (SSH(V)), a static SSH window (without ventilated air, SSH(S)), a solar control window (SC, i.e., CWO glass), an electrochromic window^[^ ^5a^ ^]^ (EC), a thermochromic window (TC, i.e., hydrogel), and a normal window (glass). The comparison between SSH(V) and SSH(S) is to show the effect of the ventilation air on the energy‐saving capacity. The optical and thermal properties of these selected windows are listed inTable S1 (Supporting Information). **Figure** **6**a–d shows the monthly HVAC energy consumptions. At Anchorage (Figure 6a), the monthly HVAC energy consumptions of SSH(V) during cold seasons are the lowest, even lower than the clear window, demonstrating the efficient synergy of solar thermal glass and the air ventilation system for space heating. At Beijing (Figure 6b), the SSH(V) performs the best during cold and hot seasons. At Hong Kong (Figure 6c) and Abu Dhabi (Figure 6d), where hot seasons dominate, the SSH(V) exhibits substantial decline of HVAC energy consumptions compared with other windows. Notably, the HVAC energy consumptions of SSH(V) from March to November are lower than those of other types of smart windows by 50% or even more, because the building cooling loadings can be remarkably reduced by integrating solar thermal glass and the air ventilation system. Furthermore, by taking the clear window as a baseline, we plotted the annual energy saving of all the cities and windows in Figure 6e. The SSH(V) shows the highest energy saving among the four cities (6.5%, 25.1%, 61.5%, and 54.9% compared with annual energy consumption of the clear window). In comparison, the thermochromic window achieves energy saving by −8.7%, 6.4%, 30.9%, and 41.1%, respectively. Note that our simulated energy‐saving data with the hydrogel thermochromic window are quite comparable to the simulation results from Zhou et al.^[^ ^21^ ^]^ We observe that the SSH(V) window delivers energy savings by around 30% and 20% higher than the most efficient hydrogel‐based thermochromic smart window^[^ ^21^ ^]^ in Hong Kong and Abu Dhabi, respectively. Besides saving the HVAC energy consumption, the SSH(V) window has a unique feature, i.e., generation of electricity, which can be utilized for indoor lighting, cooling/heating, and other applications. In Figure 6f, the electricity generation ranges from 20 MJ m^−2^ at Anchorage to 40 MJ m^−2^ at Abu Dhabi, which accounts for 4.7% and 19.1% of the annual energy savings by the SSH(V), respectively. We further calculated the power consumption of the air ventilation system based on the simulated data and found that the additional energy consumption was negligible compared to the annual energy saving and generated electricity (Note S4, Supporting Information), demonstrating that the benefit from the air ventilation system significantly overcomes its additional energy consumption.

## 3 Conclusions

In this work, we designed and fabricated a multicomponent solar harvesting window with UV and NIR dual‐band selective‐harvesting ability for maximizing solar energy utilization. The spectrally selective properties are achieved by integrating a UV‐harvesting transparent photovoltaic cell and a NIR‐absorbing transparent selective solar absorber. Because the photovoltaic only absorbs a limited portion of the solar energy (UV), a transparent selective solar absorber is designed to harvest the complementary portions of the solar energy (NIR). The integrated window shows a visible transmittance of 42% and an absorption of UV and NIR over 90%. Furthermore, we coupled an air ventilation system with the assembled window to extract the harvested thermal energy and constructed a first‐reported transparent PV/T system. We demonstrated that the SSH window produced a solar‐electricity conversion efficiency of 0.75% and a solar‐thermal conversion efficiency of 24% with a ventilated air temperature rise of 10 °C. With thermal energy harvesting by air ventilation, the total effective efficiency was estimated over 30% at a typical operating condition for building space heating applications. Our simulations suggest that the SSH window delivers an annual HVAC energy saving by up to 61.5% compared to the normal window, which is 20% higher than that of the most efficient hydrogel‐based thermochromic smart window.^[^ ^21^ ^]^ Additionally, the SSH window generates electricity that accounts for up to 19.1% of its annual energy saving amount. Our work is the first systematic attempt to integrate transparent photovoltaic and transparent solar absorber into a window. Besides its high manufacturing processability that makes it promising to commercialization, the transparent PV/T system provides unique functions of simultaneous solar regulation and solar harvesting, which has a high potential to provide a new design paradigm of zero energy buildings with minimized carbon emission.

## 4 Experimental Section

### Fabrication of a Transparent Selective Solar Absorber

Plain glass with a dimension of 300 mm × 300 mm × 6 mm) (Fuyao Glass Industry Group Co., Ltd.) was cleaned in acetone (99.5%, Scharlau Chemicals) by sonication for 20 min, followed by deionized (DI) water rinse and drying using N_2_. CWO nanoparticles of 7 nm diameter (TPK Material Solutions (Xiamen), Inc) were dissolved in ethanol (99.5%, Scharlau Chemicals), followed by sonication for 1 h and a high‐energy ball milling process for 10 h to obtain a well‐dispersed colloidal CWO solution (20 wt.%). The colloidal CWO solution was mixed into an acrylic resin with 5 wt.% Benzotriazoles UV absorbing NPs (Aladdin Bio‐Chem Technology, Shanghai). The mixing ratio of the CWO solution and the acrylic resin was 5:5 by weight. The prepared mixture was sprayed onto the plain glass substrates using a commercial ultrasonic spraying machine (UC330, Siansonic Technology Co., Ltd., Figure S2, Supporting Information).

### Preparation of a Transparent Photovoltaic Cell

One LSC‐type TPV with a dimension of 300 mm × 300 mm × 8 mm was obtained from UbiQD, Inc^[^ ^11b^ ^]^ (Figure S1, Supporting Information). The LSC‐type TPV incorporated high quantum yield (>90%), NIR‐emitting CuInS_2_/ZnS quantum dots into the polymer interlayer between two sheets of low iron float glass. The LSC‐type TPV mainly absorbed UV, transmit visible, and NIR light.

### Characterizations

The cross‐sectional image of the CWO film was obtained with a scanning electron microscopy (SEM, JSM‐7100, JEOL). The UV–vis–NIR (0.3–2.5 µm) transmittance (*T*) and absorption (*A*) spectra of the transparent solar absorber, transparent photovoltaic cell, and their integrated window were measured using a spectrometer (Lambda 950, Perkin Elmer) equipped with a 150 mm integrating sphere. For the temperature response measurement, a solar simulator (Oriel Sol2A, Newport) was used to provide standard and stable 1‐sun power (1 kW m^−2^), and T‐type thermocouples were used to measure the steady–state temperature and connected to a data acquisition device (NI 9213, National Instrument) for data recording. For the TPV electrical characterization, a Keithley 2420 SourceMeter was used to obtain *I–V* characteristics under simulated AM 1.5G solar illumination. The photovoltaic cell layouts,^[^ ^15^ ^]^ in which a matte black background was placed on the back of the TPV device, were used so that illumination from the environment or reflection could be eliminated for both *I–V* measurements.

### Field Solar Harvesting Test

Field test was conducted in Hong Kong to evaluate the solar harvesting capacity of the SSH window in a realistic outdoor environment exposure to fluctuating weather. The window was placed subject to the direct sun without any shelter. The volumetric flow rate of the air was set at 90 L min^−1^. The experiment started in 10:00 and lasted for 4.5 h. The solar irradiance, inlet and outlet air temperatures were continuously recorded, while the *I–V* curve, *V*_oc_, and short‐circuit current were measured at six time points.

### Indoor Thermal Management Test

The indoor thermal test was conducted in a control room where temperature and humidity could be precisely controlled (Figure S4, Supporting Information). Test chambers were constructed with white XPS sheets with a thickness of 5 cm. The inner space size of the chamber was 30 × 30 × 30 cm^3^ with a 30 × 30 cm^2^ opening for window installation. The volumetric flow rate of the air was set at 90 L min^−1^. A solar simulator (Oriel PVIV‐212v) was used as the light source for daytime tests. T‐type thermocouples were installed at the center of the chamber and the window surface for measuring the indoor air temperature and window surface temperature, respectively. Three windows, i.e., normal window, CWO window, and SSH window, were used with the top cover facing the solar simulator. For normal window, two layers of normal glass were used with an air gap within. For CWO window, one layer of normal glass and one layer of CWO glass were used with an air gap within. Three configurations were tested to simulate the temperature control systems in hot and cold seasons.

### Commercial Building Energy Consumption Simulations

EnergyPlus was used to evaluate the energy consumption with different windows in different climate zones. An 8 × 8 × 5 m^3^ model house was built with four 2 × 4 m^2^ windows on four walls and two 2 × 4 m^2^ skylights on the sloping roofs (Figure S5, Supporting Information). All windows had a thickness of 2.6 cm and the thermal conductivity of the windows was set as 0.5 W m^−1^ K. Climate data of Anchorage, Beijing, Hong Kong, and Abu Dhabi were selected to analyze the window performance in different latitudes. Dual set points of 20 and 26 °C were applied for the heating and cooling of the HVAC system, respectively, according to the energy‐saving suggestions from the U.S. Department of Energy.^[^ ^20^ ^]^ Since the ventilation system of SSH(V) window could not be coupled into EnergyPlus models, a heat‐transfer analysis method was developed by assuming that the solar thermal energy, which were harvested by the TSA and carried out through flowing air, was reflected by the SSH window to avoid over‐heating in the simulation (methods can be found in Note S5, Supporting Information). The calculated real‐time harvested thermal energy was applied into the room for space heating when the indoor temperature was below 20 °C, while pumped out of the house directly when the indoor temperature was above 20 °C. Consequently, the energy consumption of the SSH(V) window was obtained in the simulations.

## Conflict of Interest

The authors declare no conflict of interest.

## Author Contributions

W.L. and C.L. contributed equally to this work. W.L., C.L., and S.Y. conceived the research. W.L., C.L., and J.H. fabricated and characterized the samples and conducted the experiments. C.L. conducted energy simulations. W.L. proceeded with the energy efficiency assessment. W.L., C.L., B.H., and S.Y. analyzed the results. G.H. provided suggestions to the result analysis. W.L., C.L., B.H., and S.Y. wrote the manuscript. All authors approved the final version of the manuscript.

## Supporting information

Supporting information

## Data Availability

The data that support the findings of this study are available in the supplementary material of this article.
