# Supplementary material for: Selective Solar Harvesting Windows for Full‐Spectrum Utilization
Source: Adv Sci (Weinh). 2022 Jun 5;9(21):2201738. doi: 10.1002/advs.202201738 (PMC9313496; doi:10.1002/advs.202201738)
Supplement: Supplementary file 1 — Supporting information [file ADVS-9-2201738-s001.pdf]

Supporting Information

Selective Solar Harvesting Windows for Full-Spectrum Utilization

Weihong Li, Chongjia Lin, Gan Huang, Jun Hur, Baoling Huang, Shuhuai Yao**

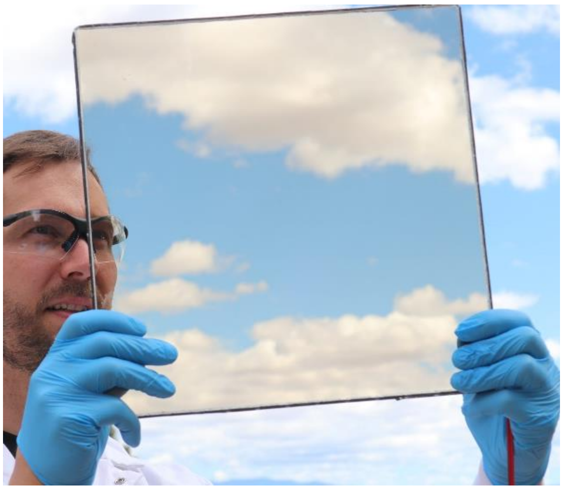

Figure S1. A commercial photovoltaic glass from UbiQD, Inc.

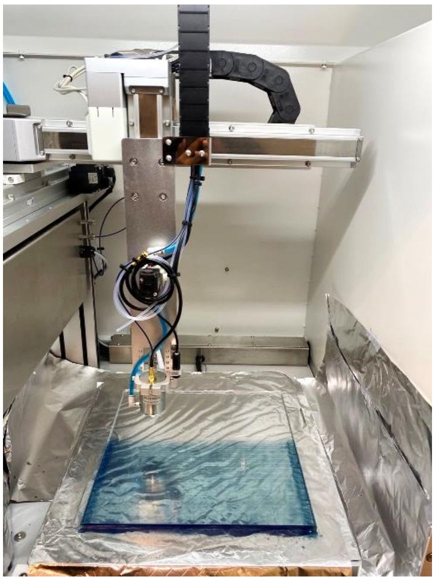

Figure S2. Fabrication of the transparent solar absorbing window using an ultrasonic spraying machine.

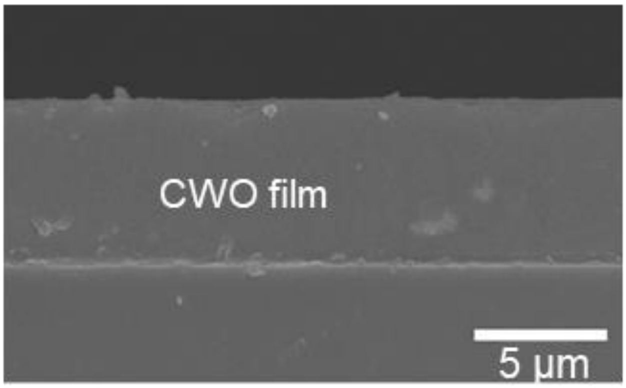

Figure S3. SEM image showing the cross-sectional view of the photothermal film.

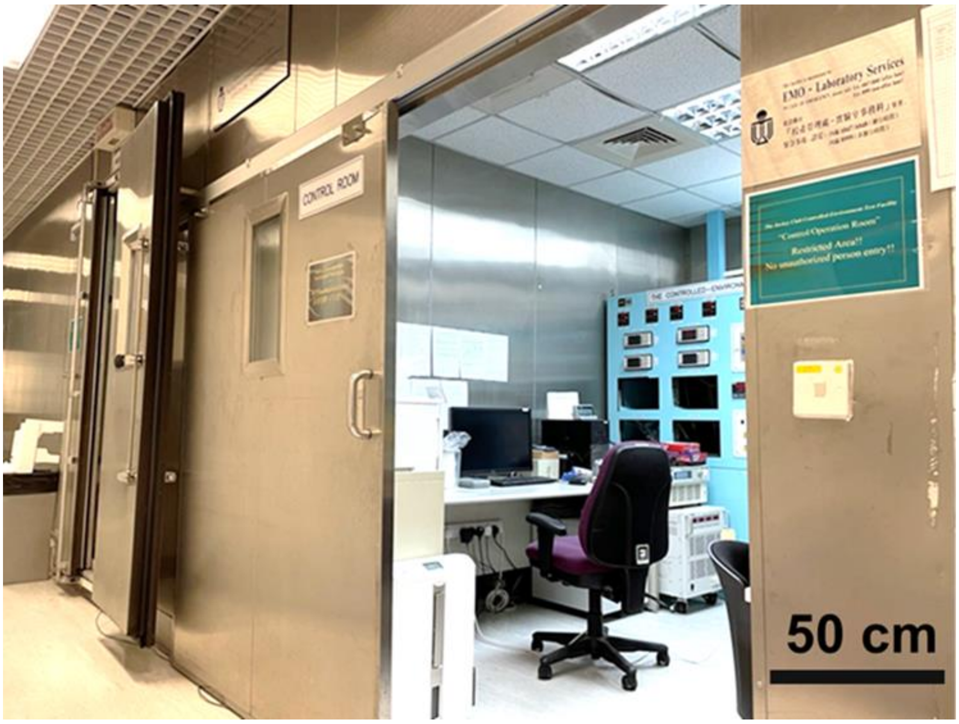

Figure S4. The industry standard environment-controlled laboratory (Hong Kong Jockey Club Controlled-Environment Test Facility) for indoor temperature regulation tests.

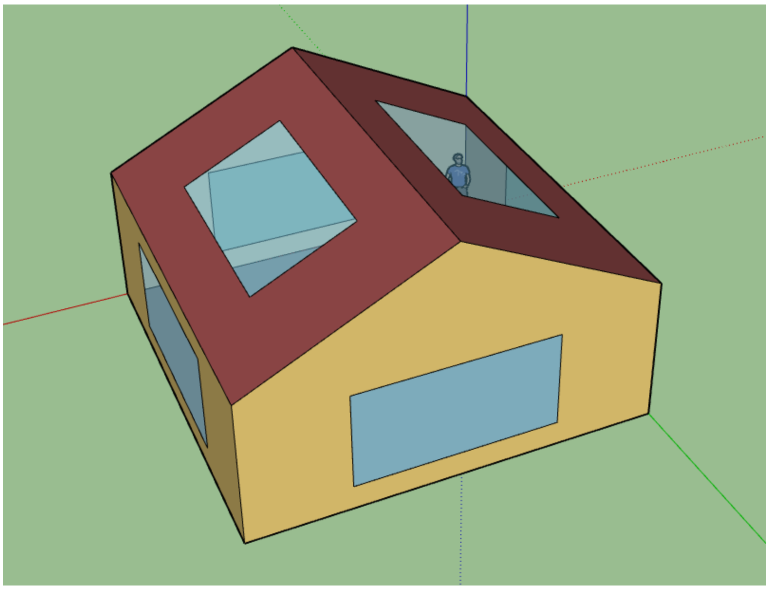

Figure S5. Model house for simulation. The area of the house is 8×8 m^2 and the height is 5 m. Four windows with a size of 4×2 m^2 in the walls and two 2.24×4 m^2 skylights on the sloping roofs are installed.

Table S1. Summary of optical and thermal properties of different windows. The data were calculated from the measured spectra and literature data.

Glazing	Solar transmittance	Visible transmittance	Solar reflectance*	Thickness (cm)	Thermal conductivity (W/m K)
Clear glass	60.8%	77.6%	7.1%	2.6	0.5
SC glass	24.7%	52.9%	5.8%	2.6	0.5
EC glass	80.0% (cold) /55.0% (hot)	88.1%	7.1%	2.6	0.5
TC glass	78.3% (cold) /9.9% (hot)	91.3% (cold) /10.5% (hot)	7% (cold) /50% (hot)	2.6	0.5
SSH (S)	17.2%	42.1%	12.8%	2.6	0.5
SSH (V)	17.2%	42.1%	12.8%+effective reflectance	2.6	0.5

* We used the same averaged solar reflectance for both front and back sides in the simulation due to the following reasons: 1) The difference of solar reflectance between front and back sides are small, since no solar reflective materials were used in the glazing layers. 2) Some windows (like the EC glass) used for the comparison come from literatures with no back-side data available. Therefore, we used the same value for both sides to deliver a fair comparison.

Supplemental Note 1. Mean solar absorptance and transmittance calculations

The mean solar absorption is calculated as[1]

$$A_{mean} = \frac{\int_{0.3\mu m}^{3.0\mu m} A(\lambda) E_{solar}(\lambda) d\lambda}{I_{solar}} \tag{S1}$$

where $A(\lambda)$, $E_{solar}(\lambda)$, and I_{solar} are the spectral absorption, spectral solar power (AM 1.5G), and total solar radiation (AM 1.5G, 1 kW m^{-2}), respectively. The visible transmittance is calculated as[2]

$$T_v = \frac{\int_{0.38\mu m}^{0.78\mu m} \phi(\lambda) T(\lambda) d\lambda}{\int_{0.38\mu m}^{0.78\mu m} \phi(\lambda) d\lambda} \tag{S2}$$

where $\varphi(\lambda)$ and $T(\lambda)$ are standard luminous efficiency for vision and spectral transmittance, respectively.

Supplemental Note 2. Calculation of average visible transmittance (AVT) and CIELAB color space

The AVT value can be determined by the ISO standard method (ISO 9050:2003) for the window applications, as given by[3]

$$AVT = \frac{\int_{380nm}^{780nm} S(\lambda)P(\lambda)T(\lambda)d\lambda}{S(\lambda)P(\lambda)d\lambda} \tag{S3}$$

where T(λ) is the transmittance of the window, S(λ) is the spectral characteristics of the light source of AM1.5G, P(λ) is the photopic response of the human eye, and dλ is the step of photon wavelength.

CIELAB color space is obtained to coordinate the color of window, per the International Commission on Illumination (CIE). The TPVs are depicted by CIE 1931 XYZ coordinates, known as XYZ tri-stimulus values[4]:

$$X = \frac{\int_{380nm}^{780nm} S(\lambda)P(\lambda)x(\lambda)d\lambda}{P(\lambda)y(\lambda)d\lambda} \tag{S4}$$

$$Y = \frac{\int_{380nm}^{780nm} S(\lambda)P(\lambda)y(\lambda)d\lambda}{P(\lambda)y(\lambda)d\lambda} \tag{S5}$$

and

$$Y = \frac{\int_{380nm}^{780nm} S(\lambda)P(\lambda)z(\lambda)d\lambda}{P(\lambda)y(\lambda)d\lambda} \tag{S6}$$

where x(λ), y(λ), and z(λ) are the color-matching functions of the CIE standard observer representing red, green, and blue visual perceptions, respectively (Figure S6). CIELAB color coordinate is calculated using these values as below, also known as chromaticity coordinates:

$$x = \frac{X}{X+Y+Z} \tag{S7}$$

$$y = \frac{Y}{X+Y+Z} \tag{S8}$$

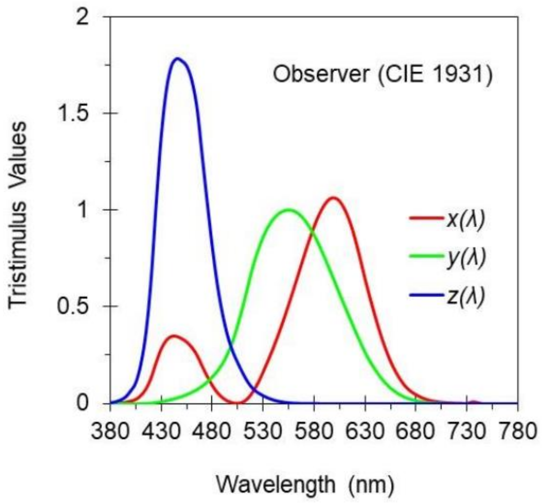

Figure S6. Color analysis. (A) Tri-stimulus values corresponding to red(x(λ)), green (y(λ)), and blue (z(λ)) color perception as a function of the photon wavelength for the observer (CIE 1931).

Supplemental Note 3. Thermal and electrical efficiency calculations

With the temperature data (inlet air temperature T_{fi} and outlet air temperature T_{fo}), massflow rate ($\dot{m}$), and solar irradiance G, we calculate the harvested thermal power w_{th} and thermal efficiency η_{th} as following:

$$w_{th} = \dot{m}c_f\left(T_{fo} - T_{fi}\right) \tag{S9}$$

$$\eta_{th} = \frac{\dot{m}c_f\left(T_{fo} - T_{fi}\right)}{GA} \tag{S10}$$

where c_f is the specific heat capacity and A is the window area. With the measured I-V curve, we calculate the generated electricity w_{ele} and electrical efficiency η_{ele} as following:

$$w_{ele} = J_{sc}V_{oc} \cdot FF \tag{S11}$$

$$\eta_{ele} = \frac{J_{sc}V_{oc} \cdot FF}{GA} \tag{S12}$$

where J_{sc} , V_{oc}, and FF are the short-circuit current density, open-circuit voltage, and fill factor of the TPV, respectively.

Supplemental Note 4. Air system pumping power calculations.

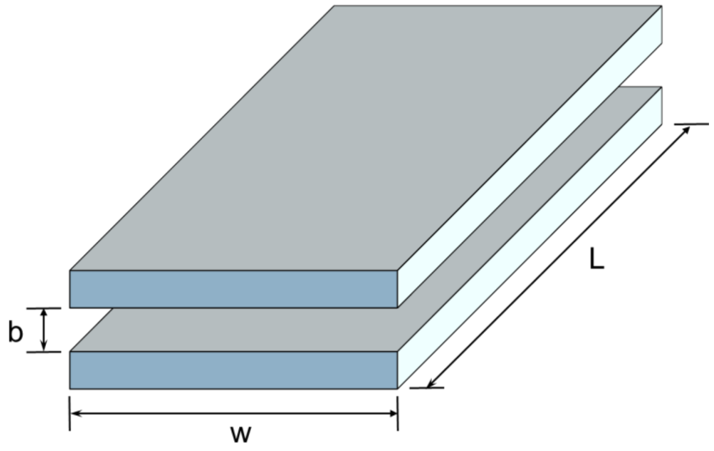

Figure S7. Geometrical parameters of two parallel plates.

Here we firstly calculate the pressure loss along two glass plates (Figure S7) and then the mechanical pumping power consumption following the method by Culham et al.[5]:

$$\Delta P = 4 \cdot f_{app} \frac{L}{D_h} \rho \frac{V^2}{2} \quad \text{(S13)}$$

The apparent friction factor, f_{app}, for hydrodynamically developing laminar flow is related to the friction factor, f, for fully developed flow and may be calculated from

$$f_{app} = \frac{\left[\left(\frac{3.44}{\sqrt{L^*}} \right)^2 + \left(f \cdot \text{Re} \right)^2 \right]^{1/2}}{\text{Re}} \quad \text{(S14)}$$

where L^* is given by

$$L^* = \frac{L/D_h}{\text{Re}} \quad \text{(S16)}$$

and the Reynolds number, Re, is given by

$$\text{Re} = \frac{\rho V D_h}{\mu} \quad \text{(S17)}$$

where μ dynamic viscosity of air. The friction factor for the fully developed laminar flow used in Eq. 13 is a function of both the aspect ratio $\lambda = b/H_f$ of the channel and the Reynolds number as given by

$$f = \left(24 - 32.527\lambda + 46.721\lambda^2 - 40.829\lambda^3 + 22.954\lambda^4 - 6.089\lambda^5 \right) / \text{Re} \quad \text{(S18)}$$

After obtaining the pressure loss within the window gap, we then calculate the mechanical pumping power of the air per kilogram:

$$\Delta e_{mech} = \frac{\Delta P}{\rho} \tag{S19}$$

Therefore, the annual mechanical pumping power of the air for the whole building windows in the simulation is

$$\Delta E_{mech} = m\frac{\Delta P}{\rho} \tag{S20}$$

and *m* is the annual mass of the consumed air. In the field test, we used volumetric air flow rate of 90 L/min for the SSH window with a dimension of 300 × 300 mm^2. In the simulated house model, we rescaled the mass flow rate for each window using the following expression

$$m = \frac{90}{1000 \times 60 \times 0.3 \times 0.01} \times 2 \times 0.01 \times 3.154e7 \cdot \rho \tag{S21}$$

where 3.157e7 is number of seconds for one year. By adopting the geometrical parameters of the simulated windows and the mass flow rate, we finally obtain the annual air pumping energy consumption is 1.06 MJ/m^2. Compared with annual energy saving and electricity generation of SSH window, the annual air pumping energy consumption is negligible. Therefore, we can conclude that the gain significant outweighs the loss.

Supplemental Note 5. Heat-transfer analysis method.

Our proposed SSH(V) window collects solar thermal energy through air ventilation and provides for indoor space heating. However, this active thermal energy harvesting behavior cannot be simulated with EnergyPlus. Therefore, we developed a heat-transfer analysis method for energy consumption simulation.

The solar absorptance of the SSH(V) window is A_o, and thereby the converted thermal energy $P_o = A_o \times G$. The converted thermal energy either dissipates to the ambient air (dissipated power: $P_d = A_d \times G$) or is collected by the ventilated air (collected power: $P_c = A_c \times G$). Thus, P_t is equal to the sum of P_d and P_c. Here we assume the equivalent solar absorptance of the SSH(V) windows for dissipated thermal energy and collected thermal energy are A_d and A_c, respectively. The following expression can be obtained:

$$P_o = A_o \times G = P_d + P_c = A_d \times G + A_c \times G \quad \text{(S22)}$$

$$A_o = A_d + A_c \quad \text{(S23)}$$

Besides, P_c / P_o is equal to the thermal efficiency η_{th}, from which we can derive A_c and A_d from η_{th} as below:

$$P_c = P_o \times \eta_{th} = A_o \times G \times \eta_{th} = A_c \times G \quad \text{(S24)}$$

$$A_c = A_o \times \eta_{th} \quad \text{(S25)}$$

$$A_d = A_o \times (1- \eta_{th}) \quad \text{(S26)}$$

The thermal efficiency η_{th} is related to both solar irradiance and air mass flow rate. In this simulation, we used a fixed air mass flow rate (100 L/min) and obtained the measured thermal efficiency 43.5%, 33.9% and 28.3% under solar irradiance of 0.4, 0.7 and 1 kW/m^2, respectively (Figure 5a). With the three measured data, we obtained a correlation function by fitting the solar radiance and thermal efficiency shown in Figure S8.

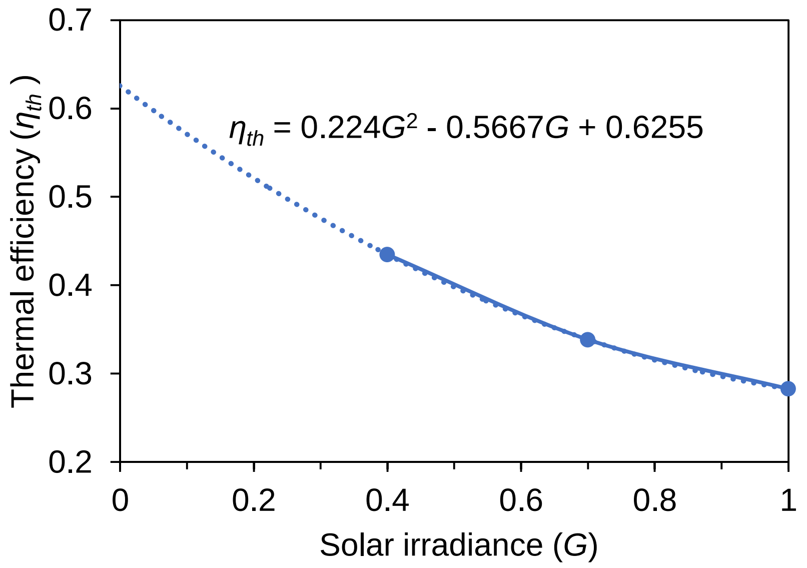

Figure S8. The function of thermal efficiency from solar irradiance

In the energy consumption simulation, we equal the solar absorptance of the SSH(V) to that of dissipated thermal energy (A_d) shown Eq. 27, and the external side solar reflectance of the SSH(V) to that of collected thermal energy (A_c) shown Equation S28. We maintained the inner side solar reflectance maintains of the SSH(V) as the measured value shown Equation S29.

$$A = A_d = A_o * (1- \eta_{th}) \quad \text{(S27)}$$

$$R_{out} = R_o + A_c = R_o + A_o * \eta_{th} \quad \text{(S28)}$$

$$R_{in} = R_o \quad \text{(S29)}$$

where A is the solar absorptance of the SSH(V) window. R_{out} and R_{in} are external and inner side solar reflectance of the SSH(V) window, respectively. A_o and R_o are the original solar absorptance and reflectance of the SSH(V) window, respectively.

We adopt the above heat transfer method to simulate the real heat dissipation behaviours by air ventilation. The energy management system (EMS) is used to input real-time solar absorptance and reflectance of the SSH(V) window calculated with Equations S27-29. Additionally, the collected thermal energy (P_c) is used for the indoor heating manually when the indoor temperature is lower than 20 °C, while it is rejected when then indoor temperature is higher than 20 °C. Consequently, we can obtain the energy consumption of the SSH(V) window in the simulations.

Supplemental References

1 Weinstein, L. A. *et al.* Concentrating solar power. *Chemical Reviews* **115**, 12797-12838 (2015).

2 Zhao, X. *et al.* Optically-switchable thermally-insulating VO2-aerogel hybrid film for window retrofits. *Appl. Energy* **278**, 115663 (2020).

3 Lee, K. *et al.* The development of transparent photovoltaics. *Cell Rep.Phys. Sci.*, 100143 (2020).

4 Patel, M., Seo, J. H., Nguyen, T. T. & Kim, J. Active energy-controlling windows incorporating transparent photovoltaics and an integrated transparent heater. *Cell Rep.Phys. Sci.* **2**, 100591 (2021).

5 Culham, J. R. & Muzychka, Y. S. Optimization of plate fin heat sinks using entropy generation minimization. *IEEE Transactions on Components and Packaging Technologies* **24**, 159-165 (2001).
